# Truth and dare: patients dare to tell the truth when using PROMs in clinical practice

**DOI:** 10.1007/s11136-024-03772-3

**Published:** 2024-10-03

**Authors:** Lotte Haverman, Michiel A. J. Luijten, Amanda L. Blackford, Kate Absolom, Ethan M. Basch, Marion A. J. van Rossum, Vivian Engelen, Martha A. Grootenhuis, Galina Velikova, Claire Snyder

**Affiliations:** 1 Amsterdam UMC Location University of Amsterdam, Emma Children’s Hospital, Child and Adolescent Psychiatry & Psychosocial Care, Meibergdreef 9, Amsterdam, The Netherlands; 2 Amsterdam Public Health, Mental Health, Amsterdam, The Netherlands; 3 Amsterdam Public Health, Digital Health, Amsterdam, The Netherlands; 4 Amsterdam Reproduction and Development, Child Development, Amsterdam, The Netherlands; 5 Amsterdam Public Health, Methodology, Amsterdam, The Netherlands; 6 Johns Hopkins Sidney Kimmel Comprehensive Cancer Center, Baltimore, MD USA; 7 Leeds Institute of Medical Research at St James’s, School of Medicine, University of Leeds, Leeds, UK; 8 University of North Carolina, Chapel Hill, NC USA; 9 Department of Pediatrics, Emma Childrens’ Hospital UMC, Amsterdam, The Netherlands; 10 Department of Pediatric Rheumatology, Amsterdam Rheumatology and Immunology Center, Location Reade, Amsterdam, The Netherlands; 11 Dutch Federation of Cancer Patients Organizations, Nederlandse Federatie Van Kankerpatiëntenorganisaties, NFK), Utrecht, The Netherlands; 12 Princess Maxima Center for Pediatric Oncology, Utrecht, the Netherlands; 13 St James’s Institute of Oncology, St James’s University Hospital, Leeds, UK; 14 Division of General Internal Medicine, Department of Medicine, Johns Hopkins University School of Medicine, Baltimore, MD USA; 15 Department of Health Policy and Management, Johns Hopkins Bloomberg School of Public Health, Baltimore, MD USA; 16 Johns Hopkins School of Medicine, 1830 East Monument, Suite 8028A, Baltimore, MD 21205 USA

**Keywords:** Patient-reported outcome measures, Clinical practice, Validity, Bias

## Abstract

**Purpose:**

As patient-reported outcome measures (PROMs) are increasingly used in clinical practice for screening, monitoring, and management, the potential for response bias has been raised (e.g., over-reporting problems for attention, under-reporting to avoid treatment changes/discontinuation). We investigated whether patients systematically bias their responses when they know clinicians will review their PROM results.

**Methods:**

We conducted secondary analyses of three experimental studies evaluating PROMs in adult and pediatric care. Prior to PROM completion, intervention group patients were informed that the results would be shown to their clinicians (“feedback” arm), whereas control group patients were told that their clinicians would not see their responses (“no feedback” arm). Independent sample *t*-tests compared the “feedback” and “no feedback” arms’ PROM scores at baseline. Effect sizes and 95% confidence intervals were estimated using Cohen’s *d* statistics with Hedges’ *g* correction, and effect sizes > 0.50 were considered clinically relevant.

**Results:**

Across the 29 domains assessed in the three studies, no between-arm differences reached an effect size of ± 0.50. Only 3/29 effect sizes exceeded ± 0.30. The confidence intervals for 14 domains included ± 0.50, with 4 favoring the “no feedback” arm and 10 favoring the “feedback” arm. Two domains reached statistical significance, one favoring the “no feedback” arm and one favoring the “feedback” arm.

**Conclusion:**

This study does not support the hypothesis that patients systematically bias their PROM responses if they know that clinicians will see their results. These findings support using PROMs in clinical practice as a valid mechanism to promote patient-centered care.

## Introduction

Patient-reported outcomes (PROs) are patients’ own reports of how they feel, function, live their lives, and survive [1, 2]. PROs add a unique perspective to more traditional measures of health such as clinician assessments and laboratory results. PROs are assessed using standardized validated PRO measures (PROMs), which are completed by patients themselves, or in some cases (e.g., young children, patients with dementia), by proxy. PROMs were initially developed to serve as endpoints in research studies evaluating the effects of interventions and are now increasingly incorporated in routine clinical practice. In the clinical practice context, PROMs are completed by patients and their data is used to inform their care. For example, PRO results can be presented in a dashboard for physicians to discuss with their patients, identify issues, monitor disease and treatment outcomes, and support shared-decision making [3].

Numerous studies have investigated the effects of PROMs in clinical practice. These studies have demonstrated that PROMs usage in adult clinical practice can enhance patient-clinician communication, improve patient satisfaction, increase efficiency, and ultimately lead to better patient outcomes, such as health-related quality of life (HRQOL), mental functioning, and even survival [4–7]. For pediatric patients, PROMs use in clinical practice has resulted in improved psychosocial outcomes and HRQOL, better detection and discussion of problems, increased treatment engagement, and enhanced patient-clinician communication [8–12]. Overall, systematic reviews [13–24] demonstrate a positive effect of PROMs on processes of care, including increased detection and diagnosis of patient issues. Recent systematic reviews, including Gibbons et al. (2021)[22] and those focusing on oncological conditions [18, 20, 21], provide moderate certainty and promising evidence of PROMs effect on outcomes such as HRQOL, (pain) symptoms, and survival.

A concern related to using PROMs in clinical practice is the potential for bias. Previous research has focused primarily on technical biases, such as non-response bias, collection method bias, fatigue bias and proxy-report bias[25]. However, a question that has been raised but, to our knowledge, remains unaddressed is whether (additional) response bias occurs if patients know their clinician is going to see their PROM results. Notably, when this topic comes up, arguments can be made for the bias going in both directions. A commonly mentioned type of bias with self-reported measures (especially on psychological functioning) is social desirability, which may result in underrepresentation of symptoms. Adolescents are especially susceptible to social desirability, which may be exacerbated when results are discussed with their healthcare providers. Another postulated example for under-reporting occurs in oncology: cancer patients may under-report their problems if they believe it will lead to treatment modification or discontinuation. It is also possible to have response bias in the opposite direction. For example, certain patients may exaggerate symptoms to get attention or additional care.

The aim of this study is to determine whether there is evidence of systematic bias in how patients report their outcomes when they are aware that their results will be shown to a clinician (and may be discussed during consultation). Because the possible bias could result in over- or underreporting, no a priori hypotheses were made.

## Methods

### Datasets

We conducted secondary analyses of three studies that evaluated the effect of using PROMs in clinical practice. In each of the studies, patients in both the intervention and control group completed PROMs, but patients in the intervention group were told that the clinician would see and/or discuss the results with the patient during the visit (“feedback” arm). Patients in the control group were told that their clinician would not see their PROM results (“no feedback” arm). By comparing the PROM scores of the feedback arms to the no feedback arms, we could investigate whether patients in the feedback arms biased their responses based on their knowledge that their clinicians would see their PROM results. These analyses only used the baseline assessments, which occurred after patients were notified about whether their PROM results would be reported to their clinicians but before the PROM intervention could have impacted the patients’ outcomes. Thus, only patients who completed baseline PROM assessments were eligible to be included in these analyses. In all three studies the no feedback and feedback arms were equivalents in terms of sociodemographic characteristics [5, 8, 9]. The single exception to this was in Haverman et al. [9], where there were significantly more adolescents (ages 13–18) in the feedback arm than in the no feedback arm.

#### Adult oncology sample—Velikova et al.[5]

The first dataset is derived from Velikova et al. [5]. This randomized controlled trial enrolled patients who were commencing cytotoxic or biologic treatment at the Leeds Cancer Centre Medical Oncology Clinic at St. James Hospital and who were expected to attend the clinic at least 3 times. Patients were randomized 2:1:1 to the intervention group (feedback arm), which involved PROM completion with feedback to clinicians; to attention-control (no feedback arm), where patients completed the PROMs but did not have their results shared with the clinician; and to the control, with no PROM completion at all. The study took place between January 2000 and July 2001. Patients were approached at the planning stage of treatment and measurement began at the start of treatment. In this study, we compared the intervention (feedback arm) to the attention-control (no feedback arm). The PROMs used included 15 domains from the European Organization for Research and Treatment of Cancer–Quality of Life Questionnaire-Core version 3.0 (EORTC-QLQ-C30)[26] and 2 domains from the Hospital Anxiety and Depression Scale (HADS)[27]. The EORTC-QLQ-C30 includes 5 function domains, 9 symptom domains, and an overall quality of life domain. Higher scores represent more of the concept being measured, so higher scores represent better function or greater symptom burden (range 0–100). The HADS has separate domains for anxiety and depression, and higher scores represent greater symptom burden (range 0–28). In total, 154 patients were included in this analysis: 47 in the no-feedback arm and 107 in the feedback arm. This secondary analysis of a de-identified dataset was not considered human subjects research and was exempt from review by the Johns Hopkins School of Medicine Institutional Review Board.

#### Pediatric oncology sample—Engelen et al. [8]

The second dataset was derived from the sequential cohort pediatric oncology study in Engelen et al. [8]. Participants were children (0–18 years) with cancer (all diagnoses) who recently completed successful treatment (0–3 months for most children; 6 months for children with stem cell transplantations) at four Dutch hospitals. PROM scores were obtained through digital questionnaires completed by the children or their parents. The results were converted into a PROfile with color-coded indicators representing the presence or absence of HRQOL problems, as well as graphical representation of the scores. Patients took part in either the no feedback arm (without PROfile information to the pediatrician—March 2006 to January 2008) or the feedback arm (provision and discussion of PROfile with pediatrician—January 2008 to November 2009), depending on the date of consultation; if a patient participated in the no feedback arm, that patient was no longer eligible to participate in the feedback arm. Randomization was not used to prevent contamination of attention to the PROMs in the no feedback arm. While various age-specific PROMs were used in the original study, this analysis focuses on the Pediatric Quality of Life Inventory (PedsQL 4.0.) Generic Core Scale [28] data. The PedsQL™ self-report form (children aged 8–18) and PedsQL parent-report form (children aged 6 and 7) were used. The PedsQL™ contains 23 items in four scales: physical health (8 items), emotional functioning (5 items), social functioning (5 items), and school functioning (5 items). A psychosocial health score—combined score of the emotional, social, and school functioning subscales—and a total scale score can be computed. Items are scored on a 5-point Likert scale from 1 ‘Never a problem’ to 5 ‘Almost always a problem’, with a one-week recall period. Answers are transformed into a 0–100 scale, with a higher score representing better HRQOL. The PedsQL was completed by 115 children: 58 in the no feedback arm and 57 in the feedback arm. Secondary data analysis of this de-identified data was deemed permissible by the Medical Ethics Testing Committee of the Amsterdam UMC.

#### Pediatric juvenile idiopathic arthritis sample—Haverman et al. [9]

The third dataset was derived from Haverman et al*. *[9]. The procedure and PROM (PedsQL 4.0.) used was the same as the Engelen et al. study [8]. The study involved children (0–18 years old) with juvenile idiopathic arthritis (JIA) who visited one of the four pediatric rheumatology centers in Amsterdam. All pediatric rheumatologists from the four participating centers were involved in the study. For this analysis, 148 children with JIA between 6 and 18 were included of whom 61 were enrolled to the no feedback arm (February 2009–April 2009) and 87 were enrolled to the feedback arm (May 2009–February 2010). Due to the chronic nature of juvenile idiopathic arthritis, the PROM intervention could have occurred at any point during treatment. Secondary data analysis of this de-identified data was deemed permissible by the Medical Ethics Testing Committee of the Amsterdam UMC.

### Analyses

As data were non-normally distributed, we applied log-10 transformation to obtain normally distributed PROM scores. We planned to use listwise deletion to handle missing data. Subsequently, we compared the PROM scores between the no feedback and feedback arms using independent sample *t*-tests. Effect sizes were calculated with the ‘effsize’ package in R using Cohen’s *d* statistic, which is the difference in the two group means divided by the pooled standard deviation. Hedge’s *g* correction was applied to account for the bias associated with small sample sizes. Corresponding 95% confidence intervals were also estimated to determine the strength of the difference, where an effect size, denoted *g*, greater than or equal to 0.50 (medium effect) was considered to be clinically relevant [29]. Because the goal of this analysis was to identify differences based on effect sizes, inferences were based primarily on whether the point estimate for the effect size reached ± 0.50 and secondarily on whether the 95% confidence intervals for the effect size included ± 0.50. Confidence intervals containing ± 0.50 identify PROM domains that could potentially show a clinically relevant difference in a different sample. On the other hand, 95% confidence intervals that do not include ± 0.50 identify PROM domains where there is likely no clinically relevant difference to be found. Finally, p-values for *t*-tests are reported descriptively; we did not adjust for multiple testing because such an adjustment would result in only larger differences in scores being considered significantly different between study arms (i.e. the effect size would have to be much higher than what we consider clinically relevant to achieve a significant p-value). All analyses were performed using R (v4.2.1)[30].

## Results

**Fig. 1:**
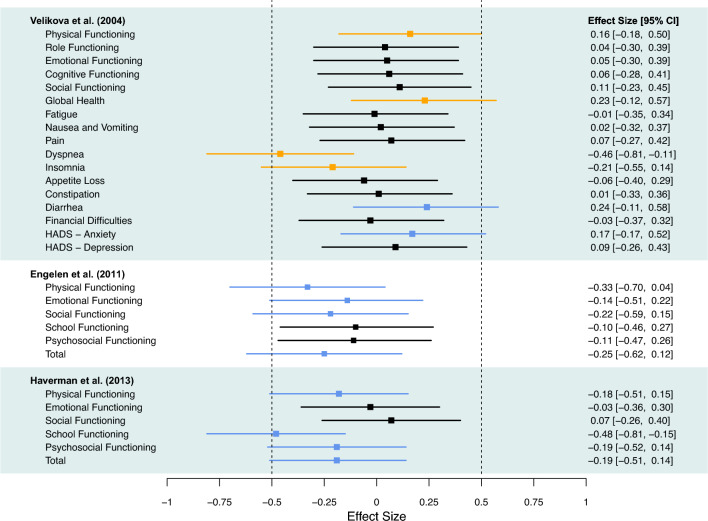
Forest plot of effect sizes (Hedges’ *g*) between “feedback” and “no feedback” arms per domain. Orange bars indicate that the “no feedback” arm reported better scores, and blue bars indicate that the “feedback” arm reported better scores (only colored for domains where the effect size confidence intervals (CI) included ± 0.50). A 95% CI that does not include the effect size of *g* =  ± 0.50, tells us that we can be fairly confident that there is no clinically relevant difference to be found

Table 1 summarizes the effect sizes comparing the intervention feedback arms to the control no feedback arms across the three study datasets. Figure 1 displays the results graphically, with orange bars indicating that the no feedback arm reported better scores on that domain and blue bars indicating that the feedback arm reported better scores on that domain. There were no missing item data in any of the three datasets.

**Table 1:** Means and standard deviations of raw scores with p-values for** i**ndependent *t*-tests for differences between intervention (“feedback”) and control (“no feedback”) arms

Velikova et al	Feedback (*n* = 107)	No feedback (*n* = 47)	*p*-value	Effect size (95% CI)
	Mean (SD)	Mean (SD)		
Physical functioning	66.2 (24.2)	69.7 (21.6)	0.31	0.16 (− 0.18, 0.50)
Role functioning	53.6 (32.22)	54.6 (30.6)	0.81	0.04 (− 0.3, 0.39)
Emotional functioning	67.0 (21.8)	71.6 (24.2)	0.81	0.05 (− 0.3, 0.39)
Cognitive functioning	77.6 (21.5)	78.0 (19.1)	0.70	0.06 (− 0.28, 0.41)
Social functioning	58.7 (30.7)	62.8 (29.7)	0.52	0.11 (− 0.23, 0.45)
Global health	52.4 (25.0)	54.3 (21.1)	0.09	0.23 (− 0.12, 0.57)
Fatigue	47.6 (28.1)	47.3 (25.4)	0.98	− 0.01 (− 0.35, 0.34)
Nausea and vomiting	17.8 (24.7)	17.0 (21.0)	0.89	0.02 (− 0.32, 0.37)
Pain	24.3 (24.5)	27.0 (26.6)	0.67	0.07 (− 0.27, 0.42)
Dyspnea	30.8 (31.0)	18.4 (27.0)	0.01	− 0.46 (− 0.81, − 0.11)
Insomnia	33.3 (32.7)	24.8 (28.2)	0.24	− 0.21 (− 0.55, 0.14)
Appetite loss	30.5 (33.7)	24.8 (26.4)	0.74	− 0.06 (− 0.40, 0.29)
Constipation	20.9 (29.2)	21.3 (29.0)	0.94	0.01 (− 0.33, 0.36)
Diarrhea	10.3 (21.2)	14.9 (23.9)	0.19	0.24 (− 0.11, 0.58)
Financial difficulties	17.5 (30.8)	14.9 (26.7)	0.88	− 0.03 (− 0.37, 0.32)
HADS—Anxiety	6.3 (3.9)	6.8 (4.1)	0.28	0.17 (− 0.17, 0.52)
HADS—Depression	5.9 (4.1)	6.0 (3.7)	0.60	0.09 (− 0.26, 0.43)

Engelen et al	Feedback (*n* = 57)	No feedback (*n* = 58)	*p*-value	Effect size (95% CI)
	Mean (SD)	Mean (SD)		
Physical functioning	74.6 (22.5)	63.7 (20.9)	0.08	− 0.33 (− 0.70, 0.04)
Emotional functioning	78.8 (19.1)	77.7 (16.7)	0.44	− 0.14 (− 0.51, 0.22)
Social functioning	84.4 (14.9)	80.2 (13.4)	0.24	− 0.22 (− 0.59, 0.15)
School functioning	73.0 (16.5)	71.0 (16.1)	0.60	− 0.10(− 0.46, 0.27)
Psychosocial functioning	78.7 (13.5)	76.3 (11.9)	0.57	− 0.11 (− 0.47, 0.26)
Total	77.3 (15.1)	71.9 (13.7)	0.19	− 0.25 (− 0.62, 0.12)

Haverman et al	Feedback (*n* = 87)	No feedback (*n* = 61)	*p*-value	Effect size (95% CI)
	Mean (SD)	Mean (SD)		
Physical functioning	69.5 (21.7)	67.1 (24.5)	0.32	− 0.18 (− 0.51, 0.15)
Emotional functioning	71.8 (22.8)	69.4 (20.6)	0.84	− 0.03 (− 0.36, 0.30)
Social functioning	79.5 (16.1)	80.3 (14.1)	0.67	0.07 (− 0.26, 0.40)
School functioning	71.7 (17.1)	63.9 (20.9)	0.01	− 0.48 (− 0.81, − 0.15)
Psychosocial functioning	74.3 (15.5)	71.2 (15.1)	0.28	− 0.19 (− 0.51, 0.14)
Total	72.7 (16.2)	69.8 (16.8)	0.26	− 0.19 (− 0.52, 0.14)

Effect sizes represent standardized differences between arms. A 95% CI that does not include the effect size of *g* =  ± 0.5, tells us that we can be fairly confident that there is no clinically relevant difference to be found

*SD* standard deviation, *CI* confidence interval

*P values for differences in mean (SD) are from two-sample *t*-tests for scores on the log scale. Effect sizes for the difference between the intervention “feedback arm” and control “no feedback arm” were calculated using Hedges' *g* for continuous scores on the log-scale. Unequal variances were assumed. For functional domains, a negative effect size indicates better scores for the feedback arm and a positive effect size indicates better scores for no feedback arm. For symptom domains, a positive effect size indicates better scores for the feedback arm and a negative effect size indicates better scores for the no feedback arm

### Adult oncology sample—Velikova et al. [5]

For the analysis of the Velikova study data, no effect sizes reached ± 0.50; however, the 95% confidence intervals for 6 of the 17 domains included 0.50. The “feedback” arm reported worse scores on 4 domains (physical function, global health, dyspnea, insomnia) but better scores on 2 domains (diarrhea, anxiety). The only difference to reach statistical significance was less severe dyspnea reported in the no feedback arm, with a p-value of 0.01 and *g* = -0.46.

### Pediatric oncology sample—Engelen et al. [8]

In Engelen’s study there were also no effect sizes that reached ± 0.50; however, the 95% confidence intervals for physical, emotional, social and total functioning did include -0.50, indicating that scores were better for the feedback arm. No differences reached statistical significance.

### Pediatric juvenile idiopathic arthritis sample—Haverman et al. [9]

In the Haverman et al. study, again, no effect sizes reached ± 0.50; however, the 95% confidence intervals for physical, school, psychosocial and total functioning did include − 0.50, favoring the “feedback” arm. The school functioning subscale differed statistically significantly between arms (*p* = 0.008, *g* = -0.48), with the feedback arm reporting better scores.

Overall, across the 29 domains assessed in the 3 studies, no domain showed differences between groups that reached an effect size of ± 0.50. Notably, 26 of the 29 effect sizes were less than ± 0.30 in magnitude. The confidence intervals for 14 domains included ± 0.50, with 4 favoring the “no feedback” arm and 10 favoring the “feedback” arm. Two domains reached statistical significance, one favoring the feedback arm (school functioning) and one favoring the no feedback arm (dyspnea). Notably, school functioning reached statistical significance in Haverman et al. 2013 but not in Engelen et al. 2011.

## Discussion

The use of PROMs in clinical practice has the potential to improve patient-clinician communication, identify issues that might otherwise go unnoticed, monitor the impact of disease and treatment, and in some cases, improve outcomes. However, these benefits rely on patients providing their true responses—regardless of whether patients’ PROM results will be shared with their provider(s). These analyses examined three independent datasets to determine whether systematic bias in PROM reporting was present based on whether patients’ results were going to be shared with their providers.

The findings from these analyses suggest that patients are not routinely changing the way they respond to PROMs based on expected review by the clinical team. The effect sizes for none of the 29 domains across the three studies reached ± 0.50, and of the 14 confidence intervals that included ± 0.50, there was no consistency in which arm was favored, with four favoring the “no feedback” arm and ten favoring the “feedback” arm. In the pediatric studies however, outcomes consistently favored the “feedback” arm indicating better scores, although these effects were not large and non-significant. Finally, only two domains were statistically significantly different (one favoring each arm), despite the conservative threshold of *p* < 0.05 across 29 tests. That being said, we strongly recommend that, whether as part of a research study or in routine practice, patients always be told whether their providers will have access to their data.

Strengths of this analysis include using three independent datasets that included both pediatric and adult patients and both cancer and arthritis populations. The studies included similar (but not identical) PROM interventions, were conducted in different years and with a different time span. In all three studies the PROM scores did not systematically differ between the “feedback” intervention groups and the “no feedback” control groups at the first assessment, when the intervention group was aware their responses would be shared with the clinician and the control group knew their responses would not be shared. In both adult and pediatric patients, and in multiple disease areas, prior knowledge of information being shared with clinicians does not seem to impact how patients report their own health.

In the Velikova study, there were no differences in the HRQOL outcomes of the RCT between the attention-control and intervention groups (the two arms included in this analysis), although there were differences in HRQOL between the intervention group and the control group with no PROM completion [5]. For the other two studies, the intervention of discussing the PROM outcomes with clinicians did significantly improve outcomes at follow-up measurements, which demonstrates that the interventions (discussing PROMs) can influence outcomes [8, 9]. We did find a statistically significant difference for the school functioning subscale of the PedsQL in the study of Haverman et al. [9]. Children reported higher (better) scores on school functioning when results were discussed with clinicians. This may be social desirability bias or children may not want to discuss how school is going with a clinician. It is possible that this is due to the distribution of age in this study, as there were significantly more adolescents (13–18 years old) in the intervention (“feedback”) group. This however does not seem to influence the outcomes on the other aspects of HRQOL and this difference in reporting on school functioning is not seen in the other study in pediatrics of Engelen et al. [8].

One of the limitations is that we could only compare the baseline PROM scores, as we would not be able to determine whether differences identified at follow-up PROM assessments resulted from bias or from the PROM intervention. While it is possible that patients would have less incentive to bias their responses at the beginning of treatment, for two of the three studies, the baseline PROM was not tied to treatment initiation. Specifically, the baseline PROM assessment was at treatment initiation only in the Velikova study; the baseline PROM assessment was after treatment in the Engelen study and at any point during treatment in the Haverman study. In addition, we could only investigate mean differences between the arms. We could not investigate the balance of under- and overreporting bias, which may have cancelled each other when analyzing at an aggregated level by using mean values. A qualitative approach and/or additional data on response biases would be required to discern the degree to which there is over- or under-reporting. However, given the amount of scales assessed in these studies and the variation of effect sizes (both negative and positive) and the lack of a bi-modal distribution in the intervention groups (Velikova et al.[5]), we do not expect this to be the case.

For the studies of Engelen and Haverman it is possible that the non-randomized sequential design of the studies may have had confounding effects on the attitudes of patients for the use of PROMs. However, in both cases randomization was not desirable as the researchers wanted to avoid contamination [8, 9]. In addition, we were unable to reliably investigate differences between age groups and between parent- and self-report or potential differences in the pediatric vs the adult group in how PROMs may be completed as sample sizes were too small. Further studies are required to investigate for example whether adolescents or parents report bias differently and what the role is of parents in completing and discussing PROMs.

Due to the small sample sizes the results should be interpreted with caution. We used multiple heterogenous studies with different measurement occasions and populations to assess clinically relevant differences by looking at effect sizes and their confidence intervals; however, it is possible that in other populations or samples these estimates could differ. Also, the relative impact of the condition for which PROMs are being reported on the patient's life may influence the risk of biased responses. For example, measuring depression in the context of a primary care visit for someone who does not experience symptoms of depression would possibly be less impacted by knowing a clinician would see the results than in the context of specialized care. Finally, our results are based on studies performed more than a decade ago. There has been increased usage of PROMs in clinical care and patients may be using PROMs differently; however, we expect this effect to be small as PROMs are still collected and discussed in a similar manner in current clinical practice.

In summary, the analyses of these three datasets do not support the hypothesis that patients systematically bias their PROM responses if they know that clinicians will see their results. These findings can reassure PRO researchers and clinicians that PROMs can be used as part of routine care, as well as for research purposes and benchmarking. As such, PROMs provide a valid and valuable mechanism to promote patient-centered care and shared-decision making.

## Data Availability

Data will be made available upon reasonable request.
